# Microwave Thermal Ablation versus Open Partial Nephrectomy for the Treatment of Small Renal Tumors in Patients Over 70 Years Old

**DOI:** 10.3390/medicina55100664

**Published:** 2019-10-01

**Authors:** Marius Anglickis, Giedrė Anglickienė, Gintarė Andreikaitė, Arminas Skrebūnas

**Affiliations:** 1 Department of Urology, Vilnius City Clinical Hospital, 10207 Vilnius, Lithuania; c.andreika@gmail.com; 2 Department of Chemotherapy, National Cancer Institute, 08406 Vilnius, Lithuania; giedre.anglickiene@gmail.com; 3 Department of Vascular Surgery, Vilnius City Clinical Hospital, 10207 Vilnius, Lithuania; arminas.skrebunas@gmail.com

**Keywords:** microwave thermal ablation, kidney cancer, glomerular filtration rate

## Abstract

*Background and objectives*: Microwave thermal ablation (MWT) is one of the treatment options for kidney cancer. However, for patients over 70 years old the safety and oncological efficacy of this treatment is still controversial. The goal of this study was to compare MWT with open partial nephrectomy (OPN) and to find out whether MWT is preferable in maintaining patient renal function and reducing the risk of postoperative complications. *Materials and Methods*: Depending on the treatment choice, all patients were divided into two groups: an MWT group and an open kidney resection (OPN) group. Data have been retrospectively collected for 7 years, starting with January 2012 up to January 2019. A total number of 33 patients with exophytic, single small renal masses were treated with either OPN (*n* = 18) or MWT (*n* = 15). All patients had histologically proven T1 kidney cancer. MWT was performed for patients who refused to have OPN or in those cases where the collecting system, renal calyx, and great vessels were free from tumor margins of more than 1 cm. *Results*: In the MWT group a median (IQR) patients’ age was 75 years (71–79) years, in the OPN group—71.5 (70–75) years, *p* = 0.005. A median (IQR) Charleston comorbidity index in the MWT group was 7.5 (5–10) and in the same way in the OPN group it was 5.22 (5–6), *p* = 0.005. A median (IQR) estimated glomerular filtration rate (eGFR) before surgery was higher in the MWT group 59.9 (49.5–73.8) mL/min/1.73 m^2^ vs. 46.2 (42.7–65.8) mL/min/1.73 m^2^ in the OPN group, *p* = 0.12. Three days following the surgery a median (IQR) eGFR was 56.45 (46.6–71.9) in MWT group mL/min/1.73 m^2^ vs. 43.45 (38.3–65) mL/min/1.73 m^2^) in the OPN group, *p* = 0.30. A median (IQR) of primary hemoglobin level was lower in the MWT group compared with the OPN group (134.5 (124–140) g/L vs. 125 (108–138) g/L), *p* = 0.41. However, after the surgery a median (IQR) lower hemoglobin level was detected in the OPN group (123.5 (111–134) g/L vs. 126 (112–135)), *p* = 0.53. The median (IQR) duration of the procedure in MWT group was shorter compared with the OPN group (26 (25–30) min vs. 67.5 (55–90) min), *p* < 0.0001. A median (IQR) hospitalization time was shorter in MWT group (3 (2–3) days vs. 89 (7–11.5) days), *p* < 0.0001. Pain by the visual analogue scale (VAS) scale the first day after surgery was significantly lower—median (IQR) in the MWT group was 2 (1–3) vs. 4 (3–6)), *p* = 0.008. Treatment failure rate was numerically higher in MWT (1/15 vs. 0/18, *p* = 0.56). *Conclusions*: Pain level on the next day after surgery, mean number of hospitalization and operation time were significantly lower in the MWT group than in the OPN group. The blood loss estimated glomerular filtration rate and oncologic data between the two groups was not statistically significant.

## 1. Introduction

The incidence of small renal masses has continued to increase over the past twenty years [1]. It is the result of widespread use of ultrasound, contrast-enhanced magnetic resonance imaging and computed tomography. Kidney resection has been a standard treatment for small renal cell carcinoma, but, frequently, open surgery is associated with a higher risk of postoperative complications (significant bleeding, requiring red blood cell transfusion, impaired renal function, etc.). One of the most common complications is deterioration of kidney function. The loss of kidney function is decreased following partial nephrectomy compared with radical nephrectomy. Studies show that 65% of patients develop grade 3 chronic kidney disease with an estimated glomerular filtration rate (eGFR) < 60 mL/min/1.73 m^2^ three years after nephrectomy [2]. Decrease in eGFR may lead to an increased risk of cardiovascular-related death in the future [3,4]. Kidney resection is the first-line treatment method for patients with small renal cell carcinoma and without serious comorbidities. The main problem is that kidney cancer is often diagnosed in elderly patients with multiple comorbidities. Those elderly patients have considerably more risk factors that could determine perioperative and postoperative complications. In particular, for patients of advanced age and those with high-risk comorbidities, including severe pulmonary and cardiovascular diseases, major operations requiring general anesthesia should be avoided. In patients with inherited renal cancer syndromes, including hereditary papillary renal carcinoma, von Hippel-Lindau syndrome, hereditary leiomyomatosis and renal cell carcinoma or Birt-Hogg-Dubé disease, renal function progressively deteriorates after multiple kidney resections. Therefore, less invasive treatments of effective cancer management and minimal loss of renal function are ideal for small kidney tumors, in patients with hereditary diseases or high-risk patients. In the past, patients with contraindications for surgery were under active observation, since the average tumor growth rate is slow (2–3 mm per year) and the metastatic potential is also rather low. The efficacies of minimally invasive therapies, including microwave thermal ablation, kidney radiofrequency ablation and cryoablation, have been reported previously. However, only a few studies have attempted to determine the effect of microwave thermal ablation treatment for elderly patients on their kidney function.

## 2. Materials and Methods

From January 2012 to January 2019, 33 patients over the age of 70 with exophytic solitary small (<4 cm) renal masses were treated with either open partial nephrectomy (OPN) or microwave thermal ablation (MWT). Preoperative characteristics of patients are shown in Table 1. All the cases in the MWT group had previously undergone kidney biopsy. MWT was done following a histological answer and during second hospitalization. Patients who had an uninformative or benign histological answer were excluded from our study. Consent for treatment was obtained in all cases following detailed information being provided to the patients on the procedure and its possible risks. The chosen treatment modality was based on tumor location and clinical evaluation of the patient. The gold standard for treatment remains surgical resection and should always be preferred when tolerable. OPN was recommended for patients without serious comorbidities and when general anesthesia was possible. Off-clamp OPN was performed in a retroperitoneal approach. MWT was performed for patients who did not want to have OPN or for patients with serious comorbidities in those cases where the urine collecting system, renal calyx, and great vessels were free from tumor margins of more than 1 cm. Other indications for ablative therapy included a solitary kidney and possible kidney disfunction following the resection. MWT was performed with a 915 MHz MicroThermXTM Microwave Ablation System generator and a single (with one 2.0 cm short tip or 4.1 cm long tip) internally cooled electrode. The selection of the tip size depended on the tumor size and its location. A short tip with 3.1 × 2.0 cm ablation zone was used for small tumors. A long tip with ablation zone of 7.0 × 6.5 cm was used for bigger tumors. Distribution of electromagnetic energy from the generator to the antenna is most commonly accomplished through a coaxial transmission line. Local anesthesia (0.5% lidocaine) and conscious sedation using 1% propofol was administered during all MWT procedures. Chest radiography, laboratory tests and CT scan were performed for each patient during a follow-up session. Parameters of technical success include therapeutic efficacy and the absence of tumor enhancement. A follow-up CT was performed 1, 3 and 6 months after MWT or OPN and then every 6 months over the years of the study. In order to determine whether there was any remnant or residual enhancement of the ablation lesion, a follow-up CT was performed after one month. The treatment was considered to be successful when no enhancement was seen on the CT. The technical success rate for surgery was defined as complete tumor ablation after the initial procedure and additional sessions within one month. New enhancing portions 3 months after the initial MWT, when nonenhancement was confirmed, or the growth of a new tumor was defined as recurrence. Statistical analysis: The Shapiro-Wilk test for normality was used to examine continuous variables. All the ordinal data was presented as an absolute number and percentage prevalence in the study population. Median scores of age, tumor size, estimated glomerular filtration rate, hemoglobin level, duration of the procedure, hospitalization time, follow-up time and other parameters in the groups were compared using the Mann–Whitney U test. Comparisons among the categorical variables were performed using the chi-square test. Interquartile range and median values were calculated to provide descriptive statistics for non-parametric tests. A *p* value <0.05 was considered statistically significant. Statistical analyses were performed by using IBM Statistical Packages for the Social Sciences (SPSS) Version 25.0 (Armonk, NY, USA, IBM Corp.).

## 3. Results

A total of 18 (54.55%) patients underwent OPN and 15 (45.45%) patients underwent MWT. Table 1 summarizes the patients’ characteristics. Patients in MWT group were older and exhibited more comorbidities than in OPN group. A median (IQR) Charleston comorbidity index in MWT group was 7.5 (5–10), in OPN group respectively it was 5.22 (5–6) years, *p* = 0.005. Pain by the visual analogue scale (VAS) scale the first day after surgery was significantly lower—median (IQR) in MWT group was 2 (1–3) vs. 4 (3–6)), *p* = 0.008. The patients’ median body mass index, the median follow-up time, baseline glomerular filtration rate and baseline serum hemoglobin did not differ significantly between the two groups. Tumor location and sex were similar in the two groups. All patients had an ASA score of 1 to 3. The greatest tumor diameter, 3.2 (2.35–3.4) cm, in the MWT group was similar to that in the OPN group, 3 (2.5–3.5) cm, *p* = 0.12). Table 2 summarizes the perioperative and postoperative characteristics. The duration of the operation, 26 (25–30) minutes, in the MWT group was significantly shorter than in the OPN group, 67.5 (55–90) minutes, *p* < 0.0001). The mean hospital stay, 3 (2–3) days, in the MWT group was significantly shorter than in the OPN group, 9 (7–11.5) days, *p* = 0.00). In OPN group, ASA 2 was for 13 patients and ASA 3 was just for one patient. At the same time, in the MWT group, ASA 2 was for nine patients and ASA 3 was for five patients (*p* = 0.62). Primary hemoglobin was lower in the MWT group when compared with the OPN group, 134.5 (124–140) g/L vs. 125 (108–138) g/L, *p* = 0.41. However, after the surgery, a lower hemoglobin level was detected in OPN group, 123.5 (111–134) g/L vs. 126 (112–135), *p* = 0.53. No recurrence or metastasis were seen in both groups. During the follow-up median period of 40 (34–47) months, radiologic evidence of incomplete ablation was found in one case (1/15, 6.66%) in the MWT group. One month after the initial MWT, a 75-year-old patient had an enhanced remaining tumor by CT. The patient underwent repeat MWT. The patient had no findings of recurrence on radiological follow-up. The OPN group had no radiologic evidence of tumor failure or recurrence. No patients in either group had minor complications such as blood transfusion, atelectasis, or wound infection. Glomerular filtration rate before surgery was higher in MWT group 59.9 (49.5–73.8) mL/min/1.73 m^2^ vs. 46.2 (42.7–65.8) mL/min/1.73 m^2^ in OPN group, *p* = 0.12. Three days after surgery, postoperative eGFR (56.45 (46.6–71.9) mL/min/1.73 m^2^) in the MWT group was similar to that in the OPN group (43.45 (38.3–65) mL/min/1.73 m^2^). Concerning the effect of both groups on preserving renal function, glomerular filtration rate according to 4-variable MDRD in the MWT group was not significantly different from that in the OPN group (*p* = 0.30).

## 4. Discussion

**Figure 1:**
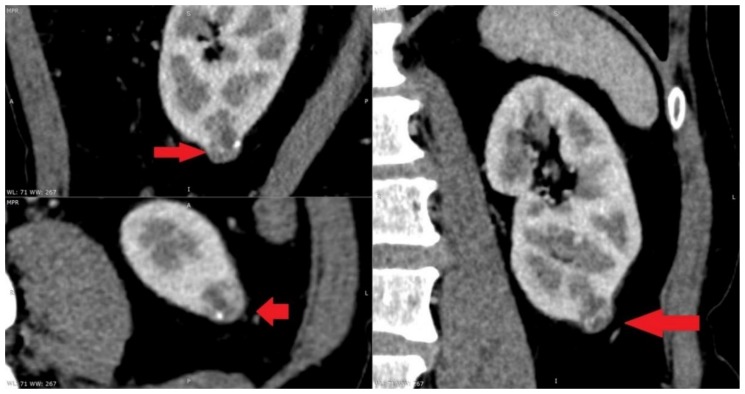
Tumor in the lower left renal pole (15 × 15 × 20 mm) before microwave thermal ablation.

**Figure 2:**
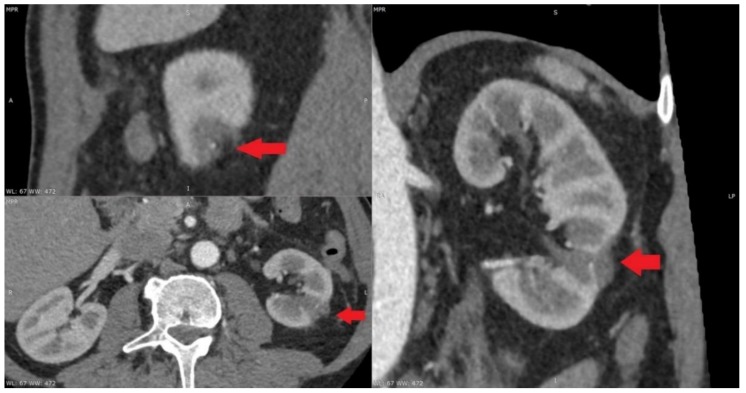
Tumor after microwave thermal ablation.

**Figure 3:**
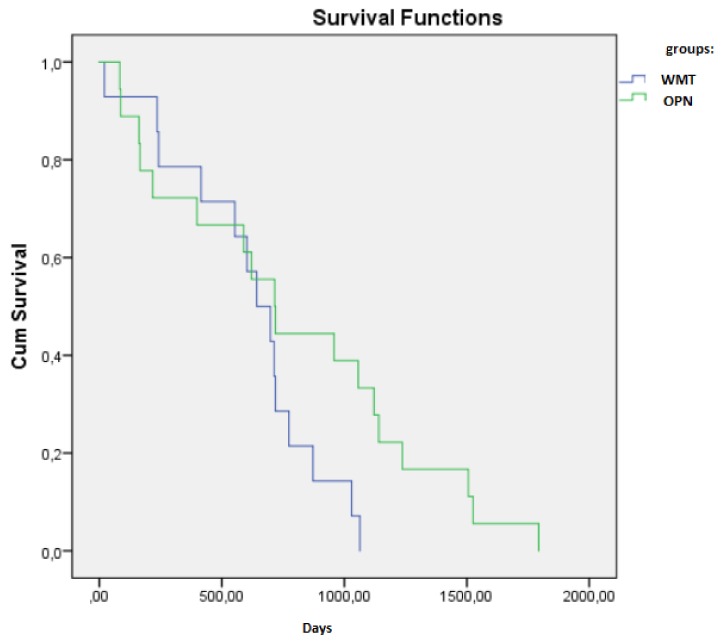
Overall survival between groups (the Kaplan-Meier method).

At present, the most widely used thermal ablative techniques are microwave ablation, radiofrequency ablation and cryoablation. The indications for MWT were considered to be comorbidity, patients’ desire for minimally invasive treatment, age, hereditary disease, solitary kidney and decreased renal function. For elderly patients, comorbid patients and patients with hereditary kidney tumors, MWT is an effective treatment modality. Microwave thermal ablation has become a viable option for the treatment of small kidney tumors (Figure 1). MWT ablation is a less invasive, less morbid treatment option (Figure 2). Microwave thermal ablation offers many benefits of other ablation techniques and offers several other advantages (faster ablation times, the ability to use multiple applicators simultaneously, higher intratumoral temperatures, less procedural pain). Excellent cancer management with rigorous follow-up periods has been reported previously in hospitals with a high volume of MWT [5,6,7]. Recently, in a retrospective review of 29 consecutive patients with a total of 30 RCC (23 T1a; 7 T1b), a technical success was achieved for 22 T1a (96%) and 7 T1b (100%) tumors using a third-generation system [8]. The present clinical study is the first to compare MWT with OPN in the treatment of elderly patients as radical surgery was often associated with an increased risk of operative and postoperative complications. Patients in MWT group were older in age. Furthermore, patients in MWT group had more comorbidities than patients undergoing open kidney resection. This study is also the first attempt to rate the pain level following the surgery. The data points to the fact that MWT treatment patients complained of less pain, thus indicating that MWT treatment is more comfortable for patients after the procedure and reduces the need for analgesics. The present study shows that MWT is an effective method for treating elderly patients with multiple comorbidities. Tumor size, RENAL nefrometry score and localization did not differ between the two analyzed groups. In our study, MWT procedure was ineffective for only 1 (6.66%) patient. This patient underwent repeated MWT. After 1 month, contrast was no longer accumulated in the tumor in control CT. Percutaneous MWT appeared to be beneficial for the majority of the patients in the present study. There was no relapse during the monitoring period. After follow-up, no distant metastases were detected. Overall survival rate was 100% (Figure 3). This suggests that MWT is an effective treatment method for T1a renal tumors. Another important aspect for older patients with multiple comorbidities is glomerular filtration rate (eGFR). Many studies have shown that chronic renal failure is associated with worse patient prognosis. All patients with stage 3 chronic kidney disease have a higher mortality and cardiovascular risk, compared with patients without chronic kidney disease. Kidney function is the main criteria for patient survival. Keeping kidney function is important because the prevalence of chronic kidney disease is estimated to be 46.8% in those older than 70 years [9]. Keeping kidney function is particularly important because renal insufficiency in older people is a strong and independent predictor of mortality. The significant correlation between predicted GFR and mortality persisted after adjustment for the effects of numerous common risk factors, including gender, smoking, hypertension, diabetes, serum cholesterol level, ischemic heart and cerebrovascular disease, BMI and physical activity [10]. In our study, baseline eGFR was lower in OPN group and its reduction after renal resection was not as dramatic as we expected. In our study, we can see that the difference in eGFR before and after MWT and OPN is reduced, but that difference is not statistically significant. Post-hoc power statistical analysis showed that the eGFR in the microwave thermal ablation group may be 13.8% lower. All patients after surgery had stage 3 kidney disease. It is expected that after minimally invasive treatment, MWT will damage kidney parenchyma less and preserve renal function. Unfortunately, our study showed that the estimated glomerular filtration rate was not better after MWT when compared with OPN. However, this may be due to the small number of patients in the study. Patients before MWT had a lower HGB. This may be related to other comorbidities. However, the difference between the groups before and after surgery was not statistically significant. Using post-hoc statistical analysis, we found a 59.6% reduction in HGB in patients after OPN. What is more interesting is that the greater half of patients who underwent MWT were at a higher risk for surgery, but the complication rate was lower and the oncological outcome was reasonable. In MWT group, patients’ ASA was worse than in OPN group, but it was not statistically significant. This shows that MWT is a suitable and safe treatment method for patients who have severe comorbidities, and for who general anesthesia and standard surgery are contraindicated. Our study concluded that patients with small kidney tumors, similar tumor location, RENAL nefrometry score and size had shorter operation times, felt less pain and shorter hospitalization times after MWT than OPN. Nevertheless, the results of this study demonstrated that MWT is superior to OPN in terms of patients’ general condition and serious comorbidities. Tumor size or location did not have any impact on changes in renal function. Though minimally invasive treatment options are determined by anatomical features (kidney tumor on the anterior surface of the kidney, tumor near to the intestine or tumor near the large renal vasculature or kidney collector) and insufficient long-term monitoring data, they are effective in kidney cancer treatment, especially for elderly patients with comorbidities. However, the present study has some limitations that warrant discussion. First, our study is a retrospective study, introducing the potential for selection bias and additional confounders. Second, there are many other MWT devices used by hospitals. Therefore, our outcomes may not be applicable universally and a more in-depth inclusive study is required.

## 5. Conclusions

The results of our study affirm that excellent therapeutic outcomes can be achieved with an MWT treatment for elderly patients by reducing operative times, hospital stays, and estimated blood loss when compared with OPN. The complication rates and procedure failure were comparable between MWT and OPN groups, there was no significant difference between those different treatment modalities. The preservation of renal function did not differ significantly between the two procedures. MWT is a minimally invasive and effective treatment modality for small kidney masses, yielding long-term oncologic outcomes equivalent to those of OPN.

## Figures and Tables

**Table 1:** Patient demographics and tumor characteristics of 33 patients.

Variable	Open Partial Nephrectomy *n* = 18 (54.55%)	Microwave Thermal Ablation *n* = 15 (45.45%)	*p* Value
Median age (years)	71.5 (IQR: 70–75)	75 years (IQR: 71–79)	0.005
Median body mass index (kg/m^2^)	25 (IQR: 23–26)	25 (IQR: 24–25)	0.42
Median tumor diameter (cm)	3 (IQR: 2.5–3.5)	3.2 (IQR: 2.35–3.4)	0.12
Median baseline glomerular filtration rate (mL/min/1.73 m^2^)	46.2 (IQR: 42.7–65.8)	59.9 (IQR: 49.5–73.8)	0.12
Median baseline hemoglobin (g/L)	134.5 (IQR: 124–140)	125 (IQR: 108–138)	0.45
Male/female	10/8	9/6	0.55
Right/left	8/10	6/9	0.76
ASA score	11/7	5/10	0.71
1	4	1	0.62
2	13	9	
3	1	5	
Tumor location			0.44
Upper	5	3	
Middle	4	8	
Lower	9	4	
Charleston comorbidity index	5.22 (IQR: 5–6)	7.5 (IQR: 5–10)	0.005
Histology			0.56
Renal cell carcinoma	15	12	
Papillary renal cell carcinoma	2	1	
Chromophobe renal cell carcinoma	1	2	
RENAL nefrometry score	5 (IQR: 4–6)	6 (IQR: 4.5–6)	0.31

**Table 2:** Perioperative and postoperative characteristics.

Variable	Open Partial Nephrectomy *n* = 18 (54.55%)	Microwave Thermal Ablation *n* = 15 (45.45%)	*p* Value
Median operation time (min)	67.5 (IQR: 55–90)	26 (IQR: 25–30)	<0.0001
Median hospital stay (d)	9 (IQR: 7–11.5)	3 (IQR: 2–3)	<0.0001
Median postoperative glomerular filtration rate (mL/min/1.73 m^2^)	43.45 (IQR: 38.3–65)	56.45 (IQR: 46.6–71.9)	0.30
VAS scale	4 (3–6)	2 (1–3)	0.008
Median postoperative hemoglobin (g/L)	123.5 (IQR: 111–134)	126 (IQR: 112–135)	0.53
Difference of GFR (mL/min/1.73 m^2^)	−0.95 (IQR: −5.1–0)	−1.4 (IQR: −4.1–0.1)	0.909
Difference of hemoglobin (g/L)	−4 (IQR: −22–1)	−1.5 (IQR: −3–1)	0.110
Failure	0	1	0.56
Median follow–up (months)	40.10 (IQR: 38–43)	40 (IQR: 34–47)	0.56
Complications (major)	0	0	
Recurrence of tumor	0	0	
Metastasis	0	0	
